# Editorial: Pharmacogenomics: From Bench to Bedside and Back Again

**DOI:** 10.3389/fgene.2022.878191

**Published:** 2022-03-14

**Authors:** Jeffrey A. Shaman, Chad A. Bousman, Christina Mitropoulou, Sandosh Padmanabhan

**Affiliations:** ^1^ Coriell Life Sciences, Philadelphia, PA, United States; ^2^ Department of Medical Genetics, University of Calgary, Calgary, AB, Canada; ^3^ The Golden Helix Foundation, London, United Kingdom; ^4^ BHF Cardiovascular Research Centre, Institute of Cardiovascular and Medical Sciences, University of Glasgow, Glasgow, United Kingdom

**Keywords:** pharmacogenomics, cytochrome P450, HLA antigens, incidental findings, clinical practice, clinical implementation, adverse drug reaction

The clinical implementation of pharmacogenomics (PGx) in the context of inpatient and outpatient medical care represents a feasible and likely influential enhancement to the therapeutic management of medications. Specifically, the utilization of genetics to identify efficacy and tolerability issues has been shown to impact economic, clinical, and humanistic outcomes that are realized by patients, healthcare providers, and payers. These positively influence healthcare’s Quadruple Aim of enhancing patient experience, improving patient outcomes, reducing costs, and improving clinician experience. The understanding of the genetic impact on pharmacokinetics and pharmacodynamics provides the foundation for the clinical utility of PGx. Layered on top are the technical, regulatory, and implementation challenges associated with providing real-world clinical decision support for personalized prescribing based on genomic data. While the recent years have delivered improvements, policy changes, and learnings from implementations of pharmacogenomics, we are now living in a unique time where technology, regulatory policy, payer policy, and medically relevant and clinically actionable science are aligned to support rational healthcare implementations. To ensure that patients are provided the opportunity to benefit from these technologies, feedback loops between molecular mechanisms, patient outcomes, and clinical implementations must be created. This Research Topic brings together seven articles focusing on PGx, affording readers the opportunity to learn about the intricacies of specific gene variants on clinical outcomes; to study the challenges, enablers, and impacts of clinical PGx implementations; to consider how to communicate often esoteric details to expand the research, clinical, and economic benefits of PGx to all stakeholders; and to contemplate the immediate and future impact of these findings.

## Emerging and Archetypal PGx Variants

The use of PGx testing in the clinic is primarily focused on genes that encode drug metabolizing enzymes in the cytochrome P450 family. Interestingly, one the most important enzymes in this family, CYP3A4, has received minimal attention and is not included within current PGx-based guidelines (Swen et al., 2011; Relling et al., 2020). Despite the discovery of a variant (*22) associated with decreased CYP3A4 enzyme activity in 2011 (Wang et al., 2011), the clinical implications of the CYP3A4*22 variant remain unclear. To address this knowledge gap, Mulder and colleagues reviewed the clinical evidence for the *22 variant’s association with the pharmacokinetics, tolerability, and efficacy of 23 drugs used to treat infectious, psychiatric, and cardiovascular diseases as well as pain and cancer (Mulder et al.). They reported that the clinical evidence supporting CYP3A4*22 based dosing is limited for most drugs but did find strong evidence supporting an association between CYP3A4*22 and the pharmacokinetics of four drugs (erythromycin, midazolam, tacrolimus, and cyclosporine). However, evidence linking CYP3A4*22 genotyping to tolerability and efficacy of these four drugs was limited, suggesting further investigation is required to establish clinical utility.

There are a few drugs, such as carbamazepine and oxcarbazepine, whose prescription can be guided by human leukocyte antigen (HLA) haplotypes to prevent severe adverse drug reactions. Buchner and co-workers investigated the analytical validity of TaqMan HLA assays for “tag” single nucleotide variants, using reference samples from diverse ancestral backgrounds (Buchner et al.). The validated TaqMan assay methodology for efficient detection of HLA haplotypes HLA-B*15:02 and HLA-A*31:01 could be readily replicated for other genotyping technologies that have the potential to identify haplotypes using single nucleotide variants.

## Clinical Uptake and Promotion of PGx Testing

Despite the availability of PGx dosing guidelines and drug labels with PGx information, the clinical uptake of PGx testing has been slow. This has been particularly the case in mental health care settings, where our understanding of the barriers as well as the enablers that contribute to the low clinical uptake are evolving. In a systematic review by Jameson and associates, nine barriers and eight enablers to the implementation of PGx testing in mental health care settings were identified and discussed (Jameson et al.). Among the barriers highlighted, cost of testing was the most frequently cited followed by physician knowledge of the evidence, and processes for integration of test result into current clinical workflows. While the most frequently cited enablers were positive perceptions of PGx testing’s role in precision prescribing, physician interest in using PGx testing, and beliefs that testing will become standard of care. As the authors aptly conclude, successful implementation of PGx testing in the mental health care setting will require thoughtful consideration of the barriers but also appropriate leveraging of enablers.

Clinical uptake and implementation of PGx information has been similarly challenging in academic health systems. In a methods article, Mroz et al. provide components and details on the steps taken to implement PGx testing in an academic health system. Included are the design of an in-house genotype and copy number assay and a reporting portal that provides a static portable document format (PDF) report. The authors emphasize a highly collaborative approach that managed the challenges and systemic barriers to implementation by coordinating the unique expertise of all local stakeholders. Education, review committees, scientific and clinical validation, and expert feedback at every point drove the success of the program and underscores the value of the bench to bedside and back paradigm highlighted in this Research Topic.

The slow adoption of pharmacogenomics in clinical settings is also experienced by many healthcare systems in low- or middle-income countries. These settings have unique challenges related to operational and economical resources, expertise, and legal, ethical, as well as local evidence-base standards that may result in initial variation in implementation of PGx in practice. However, the slow adoption of PGx in the clinic is often attributed to modest levels of PGx awareness among healthcare specialists and minimal efforts to promote PGx testing among interested stakeholders, such as healthcare professionals and biomedical scientists. In their article, Koufaki and co-workers provided a marketing perspective for the adoption of PGx in the clinic (Koufaki et al.). Particularly, these authors outlined the available marketing theories and innovations that are applied to personalized medicine interventions, which could catalyze the adoption of PGx testing in clinical practice. These authors also presented the current ethical and legal framework about genomic data and proposed ways to tackle the main concerns mentioned in the literature and to improve the marketing perspective of PGx.

Despite the challenges with clinical uptake of PGx testing, there are examples of how PGx testing can impact important clinical outcomes. In their article, David and co-workers examined the current evidence on the impact PGx testing has on hospital admissions and whether it can effectively advise towards medication changes (David et al.). The authors compared hospitalization rates and medication changes amongst PGx tested patients with patients receiving treatment-as-usual. These authors concluded that not only have medication changes occurred significantly more frequently in cases with PGx-guided treatment but also hospitalizations were significantly lower, highlighting the positive impact of PGx-guided drug prescription towards patients’ benefit and the opportunities and evidence gaps that are important when considering the introduction of PGx into health systems.

## Future Implications of PGx Results

As clinical uptake of PGx testing grows, it is prudent to consider the pleiotropic effects of pharmacogenes and the wide-reaching implications of PGx assay results and clinical reports. In a perspective article, Susanne B. Haga challenges readers to consider the identification and management of PGx secondary (i.e., incidental) findings especially in the context of both targeted and broad genetic tests like whole genome sequencing (Haga). Examples of PGx-specific genes that could also impact disease risk or phenotype highlight the need for these considerations—they are neither new, nor rare. In fact, Haga points out that, like adapting to the growing ACMG gene list, the healthcare system must be ready to deal with the ramifications of PGx testing and the incidental findings; and not just regarding host genetics. Haga suggests a logical list of steps including establishing a consensus list of secondary findings of pharmacogenes, developing patient educational materials, educating healthcare providers, and making available re-analysis and re-interpretation of variant data to reflect new evidence as it becomes available.

Recent rapid advances on multiple fronts, as demonstrated in the articles in this Research Topic, have transitioned pharmacogenomics from a niche specialist research domain to a realizable, crucial, and routine component of healthcare delivery.

## References

[B1] Relling M. V. Klein T. E. Gammal R. S. Whirl‐Carrillo M. Hoffman J. M. Caudle K. E. (2020). The Clinical Pharmacogenetics Implementation Consortium: 10 Years Later. Clin. Pharmacol. Ther. 107 (1), 171–175. 10.1002/cpt.1651 31562822 PMC6925644

[B2] Swen J. J. Nijenhuis M. de Boer A. Grandia L. Maitland-van der Zee A. H. Mulder H. (2011). Pharmacogenetics: From Bench to Byte- an Update of Guidelines. Clin. Pharmacol. Ther. 89 (5), 662–673. 10.1038/clpt.2011.34 21412232

[B3] Wang D. Guo Y. Wrighton S. A. Cooke G. E. Sadee W. (2011). Intronic Polymorphism in CYP3A4 Affects Hepatic Expression and Response to Statin Drugs. Pharmacogenomics J. 11 (4), 274–286. 10.1038/tpj.2010.28 20386561 PMC3248744

